# Impacts of Financial Stress on Mental Health and Wellbeing of Nursing Students: A Systematic Integrative Review

**DOI:** 10.1111/jan.70043

**Published:** 2025-07-01

**Authors:** Brandon W. Smith, Della Maneze, Lucie M. Ramjan, Catherine M. Stephen, Jed Montayre, Yenna Salamonson

**Affiliations:** ^1^ School of Nursing University of Wollongong Wollongong New South Wales Australia; ^2^ School of Nursing and Midwifery Western Sydney University Penrith New South Wales Australia; ^3^ Australian Centre for Integration of Oral Health (ACIOH) Ingham Institute for Applied Medical Research Liverpool New South Wales Australia; ^4^ School of Nursing The Hong Kong Polytechnic University Hung Hom Hong Kong

**Keywords:** financial stress, mental health, nursing students, pre‐registration, undergraduate, wellbeing

## Abstract

**Aim:**

To explore the direct relationship between financial stress and mental health and wellbeing of nursing students and characterise the effectiveness of available support mechanisms.

**Design:**

Systematic integrative review.

**Data Sources:**

Academic Search Complete, CINAHL, Education Research Complete, MEDLINE, ProQuest Central, PsycNET, Scopus and Web of Science were searched in January and October 2024.

**Methods:**

Studies reporting a direct relationship between financial stress and mental health and wellbeing in nursing students were included. Data related to sources of financial stress, mental health impacts, and support mechanisms were extracted, synthesised narratively, and reported thematically.

**Results:**

Findings from nine studies reveal that financial stress significantly affects nursing students' mental health and wellbeing, contributing to emotional distress and reduced quality of life. Financial stress arises from personal, academic and clinical sources, with the intensity varying based on individual demographic profiles and fluctuating throughout their educational journeys. Marginalised and underserved groups experience greater impacts due to pre‐existing disadvantages. Current support mechanisms are largely reactive, providing only short‐term relief and failing to address root causes. Additionally, students' efforts to alleviate financial stress in one domain often exacerbated it in another.

**Conclusion:**

This review highlights the multifaceted and compounding effects of financial stress on nursing students' mental health and wellbeing. Proactive strategies, including structured employment programs, embedded financial literacy education, and transparent pre‐enrolment information offer promising solutions.

**Implications for the Profession:**

While financial stress cannot be fully eradicated, targeted support for at‐risk students can mitigate its impacts, improving their mental health and educational outcomes.

**Impact:**

This review addresses the critical issue of financial stress among nursing students, highlighting its disproportionate impact on marginalised and underserved groups. It underscores the need for proactive interventions and systemic reform to improve educational experiences globally.

**Reporting Method:**

Preferred Reporting Items for Systematic Reviews and Meta‐Analyses (PRISMA) 2020 Statement.

**Patient or Public Contribution:**

No patient or public contribution.

**Trial Registration:** PROSPERO: CRD42024514262

**Summary**

- What does this paper contribute to the wider global clinical community?
  ○ Synthesises evidence on the direct and compounding effects of financial stress on nursing students' mental health and wellbeing.
  ○ Highlights the disproportionate impact of financial stress on marginalised groups within the nursing student population.
  ○ Provides evidence‐based discussion of proactive strategies to mitigate financial stress and improve educational outcomes.

## 1 Introduction

The global nursing workforce is experiencing a critical shortage, with a projected deficit of 4.5 million (Boniol et al. 2022) to 5.7 million nurses (World Health Organization 2020) by 2030. Recent factors contributing to this shortage have included COVID‐19‐related burnout, increased workloads, unsafe working conditions, staffing and equipment shortages, insufficient training, and inadequate recognition and remuneration (Buchan and Catton 2023). Increasingly, more nurses are intending to leave the profession, highlighting the urgency of addressing the widening gap between the supply of nurses and the demand for their services (International Council of Nurses 2023), and reigniting calls for effective strategies to increase the number of nurses entering the workforce.

One proposed solution is to increase the number of nursing graduates (World Health Organization 2020). Nevertheless, this seemingly straightforward approach to bolstering the supply of qualified nurses is fraught with challenges. Current global efforts to increase completion rates are largely inadequate (Buchan and Catton 2023). Concurrently, high attrition rates continue to undermine these efforts and drive the development of retention initiatives, such as the Reducing Pre‐registration Attrition and Improving Retention (RePAIR) project in the United Kingdom (Health Education England 2018) and retention models like the Nursing Universal Retention and Success (NURS) model (Jeffreys 2022). Yet, attrition rates remain alarmingly high in some regions, ranging from 7.3% to 50% depending on the context and definition of attrition (Canzan et al. 2022; Chan et al. 2019; Mazzotta et al. 2024). These efforts reflect an urgent need to support nursing students as they progress through education and into the profession, recognising that the sustainability of the future workforce depends on the wellbeing and retention of this group.

Key factors impacting outcomes for students include the challenge of adapting to unfamiliar academic and social environments, the loss of existing support networks, the need for increased personal autonomy and balancing study, work, and personal commitments (Mulvogue et al. 2023). Such persistent stress affects students' ability to focus on their studies (Labrague et al. 2017; Timmins and Kaliszer 2002), which can adversely impact their academic performance and, ultimately, progression. Among these stressors, financial strain is a particularly insidious and debilitating factor for nursing students, present throughout their academic journey and frequently cited as a reason for considering withdrawal (Health Education England 2018). Everyday living expenses are compounded by the costs of education, including tuition fees and purchasing learning materials and equipment (Moore et al. 2021). For nursing students, the financial burden is further exacerbated by the requirement to complete substantial unpaid clinical placements, which are mandatory for professional registration (Evans and Bonner 2023), leaving little time for paid employment while studying. Marginalised and underserved groups, including those from lower socioeconomic backgrounds, international students, mature‐age students, and single parents often experience greater challenges (Brough et al. 2014) due to limited access to financial resources and additional financial responsibilities (Department of Education 2024). As these groups become more prominent in nursing cohorts (Jefferys 2012), the prevalence and complexity of financial stress is amplified.

This challenge is not new; financial stress among nursing students has been documented for over four decades (see Dean 2023; Longmore 2023, 2024; Morton 1983). More recently, discussions have intensified around the toll of unpaid clinical placements, often termed “placement poverty” (see Fedele 2024; Lloyd 2024). Despite the vast editorial commentary, empirical research exploring the direct relationship between financial stress and the mental health and wellbeing of nursing students remains scarce, and evidence from the broader literature further emphasises the knowledge gap. For instance, a rapid review by McCloud and Bann (2019) identified an association between financial stress and mental health outcomes among higher education students; however, the strength of these relationships was found to be tenuous. Similarly, a scoping review on financial hardship among midwifery students in Australia reported that “students experience a range of physical and psychological health issues related to the financial pressures they face” (Moran et al. 2024, 7); however, what constitutes “psychological health issues” was not specified, and only two of the eight sources included in the review were primary studies.

The disparity between the well‐documented experiences of financial stress among university students (Brough et al. 2014; Moore et al. 2021) and the limited empirical evidence on its specific impact on the mental health and wellbeing of nursing students highlights a critical knowledge gap. Bridging this gap would provide valuable insight to guide policy development for the future of nursing education and workforce planning. This review therefore examines the direct relationship between financial stress and the mental health and wellbeing of nursing students, offering a focused synthesis of the existing empirical literature. In doing so, it advances understanding of a key contributor to underperformance, attrition, and delayed completion during the formative years of a nursing career.

## 2 Aim

The aim of this review was to explore the direct relationship between financial stress and the mental health and wellbeing of nursing students. This was guided by the primary research question: How does financial stress impact the mental health and wellbeing of nursing students? To address this overarching question, this review focused on three sub‐questions:

1. What are the sources of financial stress for nursing students?
2. What are the characteristics of personal, social, and systemic supports available to nursing students experiencing financial stress?
3. How effective are personal, social, and systemic supports in reducing adverse impacts of financial stress?

## 3 Methods

### 3.1 Design

An integrative review framework was chosen to address the complex and multifactorial relationship between financial stress and mental health and wellbeing. This approach accommodates diverse research methodologies and perspectives, enabling the synthesis of both quantitative and qualitative data for a more comprehensive understanding of the phenomenon of interest (Whittemore and Knafl 2005). To ensure rigour and enhance the systematic nature of the review process, this review was registered with PROSPERO (CRD42024514262), guided by the JBI Manual for Evidence Synthesis (Aromataris and Munn 2020), and reported using the Preferred Reporting Items for Systematic Reviews and Meta‐Analyses (PRISMA) 2020 Statement (Page et al. 2021).

### 3.2 Search Methods

This review employed a three‐phase search strategy (Lockwood et al. 2024). In the first phase, preliminary searches were conducted using Google Scholar to identify relevant search terms from existing literature. The final search strategy was developed in collaboration with a university librarian and structured around four concepts: “university students”, “financial stress”, “mental health and wellbeing”, and “nursing”. Within each concept, synonymous keywords were combined using the OR operator, and the four concepts were then combined using the AND operator. Lemmatisation techniques were applied. For example, the truncation “nurs*” was used to capture variations such as “nurse”, “nurses”, and “nursing”. Field options in each database restricted the search to the title, abstract, and keywords.

In the second phase, eight databases were accessed on 23 January 2024: Academic Search Complete, CINAHL, Education Research Complete, MEDLINE, ProQuest Central, PsycNET, Scopus, and Web of Science. Searches were limited to English‐language publications with no additional filters applied. To ensure currency, all searches were re‐run on 31 October 2024 to capture newly published studies. The complete search strategy is provided in Table 1.

**TABLE 1:** Concept map and search strategy.

Concept 1: University students	Concept 2: Financial hardship	Concept 3: Mental health and wellbeing	Concept 4: Nursing	Database	Results
Baccalaureate OR undergraduate student OR university student OR college student OR tertiary student OR tertiary education OR tertiary institution OR post‐secondary student OR post‐secondary education OR higher education OR pre‐registration	Debt OR indebted* OR poverty OR impoverish* OR low income OR out‐of‐pocket OR “education cost” OR “financial burden” OR “financial consequence” OR “financial difficulty” OR “financial difficulties” OR “financial distress” OR “financial hardship” OR “financial insecurity” OR “financial precarity” OR “financial problem” OR “financial strain” OR “financial stress” OR “financial toxicity” OR “economic burden” OR “economic consequence” OR “economic difficulty” OR “economic difficulties” OR “economic distress” OR “economic hardship” OR “economic insecurity” OR “economic precarity” OR “economic problem” OR “economic strain” OR “economic stress”	mental OR psychological OR anxiety OR depression OR stress OR distress OR wellbeing OR well‐being OR “well being”	nurs*	Academic Search Complete	415
baccalaureate OR undergraduate student OR university student OR college student OR tertiary student OR tertiary education OR tertiary institution OR post‐secondary student OR post‐secondary education OR higher education OR pre‐registration	Debt OR indebted* OR poverty OR impoverish* OR low income OR out‐of‐pocket OR “education cost” OR “financial burden” OR “financial consequence” OR “financial difficulty” OR “financial difficulties” OR “financial distress” OR “financial hardship” OR “financial insecurity” OR “financial precarity” OR “financial problem” OR “financial strain” OR “financial stress” OR “financial toxicity” OR “economic burden” OR “economic consequence” OR “economic difficulty” OR “economic difficulties” OR “economic distress” OR “economic hardship” OR “economic insecurity” OR “economic precarity” OR “economic problem” OR “economic strain” OR “economic stress”	mental OR psychological OR anxiety OR depression OR stress OR distress OR wellbeing OR well‐being OR “well being”	Nurs*	PsycINFO	321
baccalaureate OR undergraduate student OR university student OR college student OR tertiary student OR tertiary education OR tertiary institution OR post‐secondary student OR post‐secondary education OR higher education OR pre‐registration	Debt OR indebted* OR poverty OR impoverish* OR low income OR out‐of‐pocket OR “education cost” OR “financial burden” OR “financial consequence” OR “financial difficulty” OR “financial difficulties” OR “financial distress” OR “financial hardship” OR “financial insecurity” OR “financial precarity” OR “financial problem” OR “financial strain” OR “financial stress” OR “financial toxicity” OR “economic burden” OR “economic consequence” OR “economic difficulty” OR “economic difficulties” OR “economic distress” OR “economic hardship” OR “economic insecurity” OR “economic precarity” OR “economic problem” OR “economic strain” OR “economic stress”	mental OR psychological OR anxiety OR depression OR stress OR distress OR wellbeing OR well‐being OR “well being”	nurs*	CINAHL	166
baccalaureate OR undergraduate student OR university student OR college student OR tertiary student OR tertiary education OR tertiary institution OR post‐secondary student OR post‐secondary education OR higher education OR pre‐registration	debt OR indebted* OR poverty OR impoverish* OR low income OR out‐of‐pocket OR “education cost” OR “financial burden” OR “financial consequence” OR “financial difficulty” OR “financial difficulties” OR “financial distress” OR “financial hardship” OR “financial insecurity” OR “financial precarity” OR “financial problem” OR “financial strain” OR “financial stress” OR “financial toxicity” OR “economic burden” OR “economic consequence” OR “economic difficulty” OR “economic difficulties” OR “economic distress” OR “economic hardship” OR “economic insecurity” OR “economic precarity” OR “economic problem” OR “economic strain” OR “economic stress”	mental OR psychological OR anxiety OR depression OR stress OR distress OR wellbeing OR well‐being OR “well being”	Nurs*	Education Research Complete	73
baccalaureate OR undergraduate student OR university student OR college student OR tertiary student OR tertiary education OR tertiary institution OR post‐secondary student OR post‐secondary education OR higher education OR pre‐registration	Debt OR indebted* OR poverty OR impoverish* OR low income OR out‐of‐pocket OR “education cost” OR “financial burden” OR “financial consequence” OR “financial difficulty” OR “financial difficulties” OR “financial distress” OR “financial hardship” OR “financial insecurity” OR “financial precarity” OR “financial problem” OR “financial strain” OR “financial stress” OR “financial toxicity” OR “economic burden” OR “economic consequence” OR “economic difficulty” OR “economic difficulties” OR “economic distress” OR “economic hardship” OR “economic insecurity” OR “economic precarity” OR “economic problem” OR “economic strain” OR “economic stress”	mental OR psychological OR anxiety OR depression OR stress OR distress OR wellbeing OR well‐being OR “well being”	Nurs*	MEDLINE	640
Noft (baccalaureate OR undergraduate student OR university student OR college student OR tertiary student OR tertiary education OR tertiary institution OR post‐secondary student OR post‐secondary education OR higher education OR pre‐registration)	Noft (debt OR indebted* OR poverty OR impoverish* OR low income OR out‐of‐pocket OR “education cost” OR “financial burden” OR “financial consequence” OR “financial difficulty” OR “financial difficulties” OR “financial distress” OR “financial hardship” OR “financial insecurity” OR “financial precarity” OR “financial problem” OR “financial strain” OR “financial stress” OR “financial toxicity” OR “economic burden” OR “economic consequence” OR “economic difficulty” OR “economic difficulties” OR “economic distress” OR “economic hardship” OR “economic insecurity” OR “economic precarity” OR “economic problem” OR “economic strain” OR “economic stress”)	Noft (mental OR psychological OR anxiety OR depression OR stress OR distress OR wellbeing OR well‐being OR “well being”)	Noft (nurs*)	ProQuest central	1177
TITLE‐ABS‐KEY (baccalaureate OR “undergraduate student” OR “university student” OR “college student” OR “tertiary student” OR “tertiary education” OR “tertiary institution” OR “post‐secondary student” OR “post‐secondary education” OR “higher education” OR pre‐registration)	TITLE‐ABS‐KEY (debt OR indebted* OR poverty OR impoverish* OR low income OR out‐of‐pocket OR “education cost” OR “financial burden” OR “financial consequence” OR “financial difficulty” OR “financial difficulties” OR “financial distress” OR “financial hardship” OR “financial insecurity” OR “financial precarity” OR “financial problem” OR “financial strain” OR “financial stress” OR “financial toxicity” OR “economic burden” OR “economic consequence” OR “economic difficulty” OR “economic difficulties” OR “economic distress” OR “economic hardship” OR “economic insecurity” OR “economic precarity” OR “economic problem” OR “economic strain” OR “economic stress”)	TITLE‐ABS‐KEY (mental OR psychological OR anxiety OR depression OR stress OR distress OR wellbeing OR well‐being OR “well being”)	TITLE‐ABS‐KEY (nurs*)	Scopus	53
TS (baccalaureate OR undergraduate student OR university student OR college student OR tertiary student OR tertiary education OR tertiary institution OR post‐secondary student OR post‐secondary education OR higher education OR pre‐registration)	TS (debt OR indebted* OR poverty OR impoverish* OR low income OR out‐of‐pocket OR “education cost” OR “financial burden” OR “financial consequence” OR “financial difficulty” OR “financial difficulties” OR “financial distress” OR “financial hardship” OR “financial insecurity” OR “financial precarity” OR “financial problem” OR “financial strain” OR “financial stress” OR “financial toxicity” OR “economic burden” OR “economic consequence” OR “economic difficulty” OR “economic difficulties” OR “economic distress” OR “economic hardship” OR “economic insecurity” OR “economic precarity” OR “economic problem” OR “economic strain” OR “economic stress”)	TS (mental OR psychological OR anxiety OR depression OR stress OR distress OR wellbeing OR well‐being OR “well being”)	TS (nurs*)	Web of Science	299
				Total	3144

In the third phase, two authors (BWS and YS) independently conducted forward and backward citation searches. The forward search involved screening subsequent citations of the included studies using Google Scholar, and the backward search involved screening the reference list of each included study. In addition to these traditional methods, this review incorporated a novel verification step that leveraged artificial intelligence (AI) tools to triangulate and confirm the comprehensiveness of the search strategy. BWS used Semantic Scholar [https://www.semanticscholar.org/] to replicate the forward and backward searches, then used Litmaps [https://www.litmaps.com/] to retrieve the 100 “Top Shared Citations & References”. These were screened against the eligibility criteria, with 97 references excluded by title and abstract. The three remaining references had already been identified in the initial searches and were again excluded for lacking a mental health and wellbeing component, reporting non‐specific “stress”, and a non‐nursing/blended cohort.

Concurrently, YS used ResearchRabbit [https://www.researchrabbit.ai/] to verify the accuracy of the forward searches. No discrepancies or additional eligible studies were identified. While no new inclusions resulted from the AI tools, they provided a useful layer of verification and reinforced the thoroughness of the search strategy.

### 3.3 Inclusion and Exclusion Criteria

#### 3.3.1 Population

The population of interest was nursing students enrolled in an accredited higher education programme that leads to professional registration as a Registered Nurse, regardless of entry pathway. Studies involving non‐nursing students, mixed disciplines, programmes outside higher education settings, or programmes not leading to professional registration were excluded.

#### 3.3.2 Primary Exposure

The primary exposure of interest was financial stress, defined in this review as a situation characterised by a sense of deficiency arising from a discrepancy between available financial resources and financial obligations (Wuth and Cismaru 2021). This broad operational definition was adopted to accommodate variation in how financial stress was measured across studies. Examples of quantitative measurements included income‐to‐expense ratios, comparisons to low‐income or poverty thresholds, or validated tools. Qualitative financial stress was identified through participants' subjective accounts.

#### 3.3.3 Secondary Exposure

The secondary exposure of interest was support mechanisms, defined in this review as any strategies or interventions aimed at alleviating financial stress or mitigating its impact on mental health and wellbeing. Supports were categorised into three types: (1) personal, referring to self‐initiated behaviours; (2) social, referring to assistance from family, friends, and peers; and (3) systemic, referring to institutional or government‐provided resources. As a secondary exposure, the presence or absence of support mechanisms was not used as an inclusion criterion. When reported, data on each type of support was extracted and analysed to evaluate its role in shaping the relationship between financial stress and mental health and wellbeing.

#### 3.3.4 Outcome

The outcome of interest was mental health and wellbeing, defined according to the World Health Organization (2021, 1) as “a state… in which the individual [realises their] own abilities, can cope with the normal stresses of life, can work productively and fruitfully, and is able to make a contribution to [their] community”. To be included, studies had to report a direct relationship between financial stress and an alteration in mental health and wellbeing. The term “alteration” was deliberately chosen for inclusivity, encompassing both positive and negative changes. Outcomes could be measured quantitatively using validated tools assessing symptomatology, prevalence, incidence, or functional impacts of mental health conditions; or qualitatively through participants' subjective accounts. Studies that examined both the primary exposure and the outcome but reported no association were excluded to maintain a focused exploration of the impacts of financial stress on mental health and wellbeing. Additionally, studies that referred only to generic “stress” were excluded due to a lack of conceptual specificity required for addressing this review's questions.

#### 3.3.5 Study Design

Only peer‐reviewed primary research studies employing qualitative, quantitative, or mixed‐methods designs and published in English were included. Unpublished or grey literature, secondary research, editorials, discussion papers, opinion pieces, and theses or dissertations were excluded.

### 3.4 Search Outcome

All references were imported into EndNote version 20.6 for duplicate removal and screening against the eligibility criteria. Two authors (BWS and YS) independently screened titles and abstracts, and two authors (BWS and DM) independently conducted full‐text screening.

### 3.5 Bias Assessment and Quality Appraisal

Risk of bias and methodological quality were assessed using the Joanna Briggs Institute *Checklist for Analytical Cross‐Sectional Studies* (Moola et al. 2024) or *Checklist for Qualitative Research* (Lockwood et al. 2015), depending on the design of each included study. All studies were appraised independently by two authors (BWS and DM) and disagreements were resolved following independent review by a third author (YS) to achieve consensus.

### 3.6 Data Abstraction

A data extraction table was developed and pilot tested by one author (BWS) using the included studies, then reviewed by two other authors (YS and DM). Following team discussion and consensus on the fields, final data extraction was completed by one author (BWS) and cross‐checked for accuracy by two authors (YS and DM). Data were extracted on the following variables: author, year, country, aim, study design, sample characteristics, sources of financial stress, alterations to mental health and wellbeing, and the presence, type, and effectiveness of supports.

### 3.7 Data Synthesis

Due to the methodological heterogeneity of the included studies, a narrative synthesis was conducted. This approach facilitated the integration of data from diverse study designs, allowing for the transformation of quantitative data into qualitative descriptions suitable for thematic analysis (Frantzen and Fetters 2016). Theme development was informed by repeated reading, coding of extracted data and comparison of concepts across studies (Braun and Clarke 2022). Patterns were identified and synthesised into three overarching themes: (1) impacts on mental health and wellbeing, (2) sources of financial stress, and (3) sources and effectiveness of support.

## 4 Results

### 4.1 Search Results

The initial search, performed on 23 January 2024, identified 3144 references. After removing 1075 duplicates, 2069 references were screened by title and abstract, excluding 1999. Full‐text screening of the remaining 70 references excluded 64. One study meeting the eligibility criteria was identified through backward searches, resulting in seven studies included from the first search.

The second search, performed on 31 October 2024, identified 3570 references. From these, all 428 references published in 2024 were isolated, with 175 duplicates and 11 previously screened references removed. Title and abstract screening of the remaining 242 references excluded 236. Six full texts were screened, with five excluded. Backward searches identified one additional eligible study, resulting in two studies included from the second search.

**FIGURE 1:**
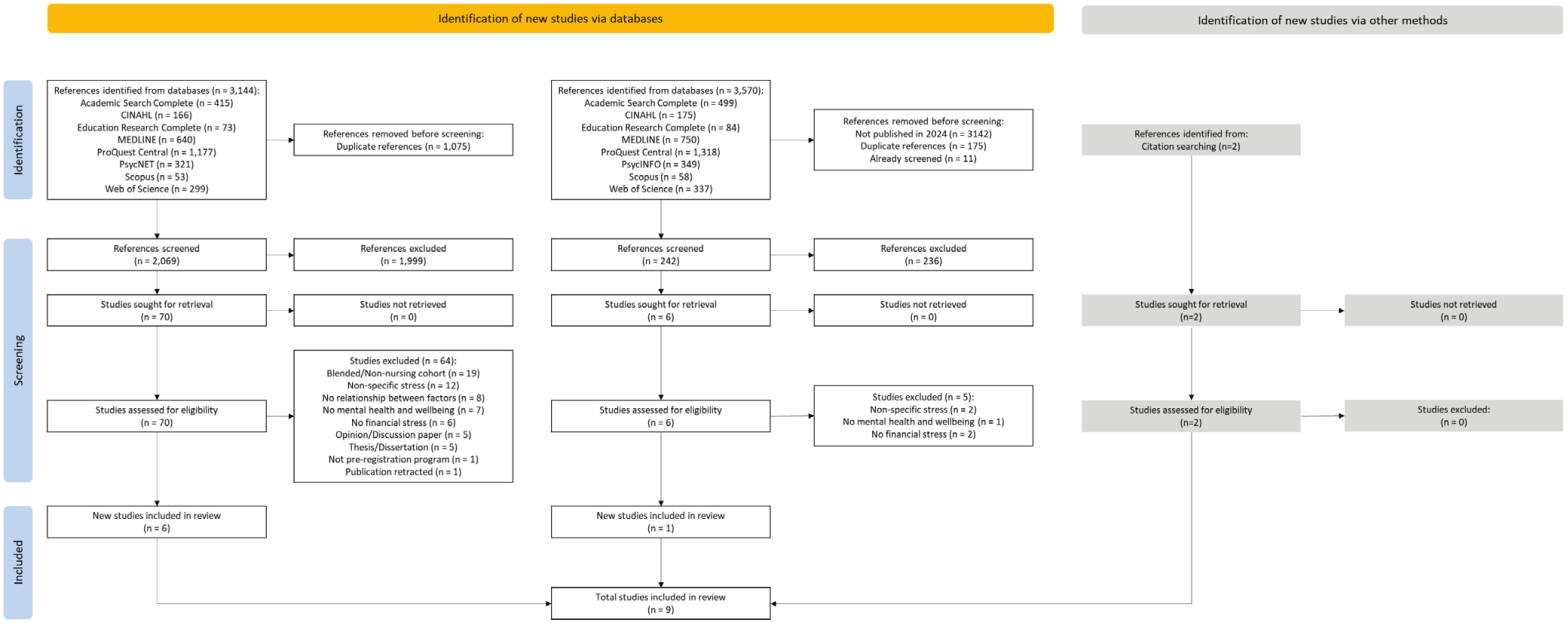
PRISMA flow diagram.

In total, nine studies are included in this review: seven from the first search and two from the second search. An overview of this process and the reasons for full‐text exclusion are provided in Figure 1.

### 4.2 Study Characteristics

The nine studies included in this review span two decades, with publication dates between 2004 (Kevern and Webb 2004) and 2024 (Tiga‐Loza et al. 2024). Their geographic distribution was diverse, highlighting the global relevance of financial stress for nursing students; four studies were conducted in Australia (Andrew et al. 2022; Ogunsiji and Wilkes 2004; Usher et al. 2022, 2005), and the remaining studies originated from Brazil (Santos et al. 2020), Colombia (Tiga‐Loza et al. 2024), England (Kevern and Webb 2004), Hong Kong, China (Cheung et al. 2016) and Portugal (Silva et al. 2021). In relation to research design, four of the studies employed qualitative methodologies using interviews (Andrew et al. 2022; Ogunsiji and Wilkes 2004; Usher et al. 2005) and focus groups (Kevern and Webb 2004) to explore the experiences of specific demographic groups within the undergraduate nursing cohort, including Indigenous students (Usher et al. 2005), single mothers (Ogunsiji and Wilkes 2004), women with family responsibilities (Andrew et al. 2022), and women aged over 25 years (Kevern and Webb 2004). Four quantitative studies used a cross‐sectional survey design to measure elements of mental health and wellbeing—Cheung et al. (2016) explored the prevalence of depression, anxiety and stress, Santos et al. (2020) explored the correlation between sociodemographic and behavioural variables and sleep quality, Silva et al. (2021) explored the relationship between psychological wellbeing and coping strategies, and Tiga‐Loza et al. (2024) explored the factors associated with mental health disturbances. The study by Usher et al. (2022) specifically focused on financial stress but combined quantitative and qualitative questions to explore experiences during clinical placements. A summary of the studies included in this review is provided in Table 2.

**TABLE 2:** Summary table of studies included in this review.

Author, year, country, quality score	Aim, study design, sample characteristics	Sources of financial stress, alterations to mental health and wellbeing	Personal supports and their effectiveness	Social supports and their effectiveness	Systemic supports and their effectiveness
Andrew et al. (2022) **Country:** Australia **Quality score:** 80%	**Aim:** To explore the experiences of women with family responsibilities studying nursing **Study design:** Qualitative guided by Gadamer's hermeneutic philosophy **Sample characteristics:** 29 women enrolled in a Bachelor of Science (Nursing) degree at a Western Australian university	**Sources of financial stress:** Casual or low‐income employment Lost income during placement Loss of second income following relationship breakdown; and Household and personal expenses **Alterations to mental health and wellbeing:** Students reported fatigue and exhaustion due to long work hours	Students used leave entitlements to offset lost income during placements and worked additional hours around placements, reducing available time for study	Students received instrumental support from partners (often FIFO workers) but retained full responsibility for domestic duties Living with parents also provided instrumental support through reduced housing costs, but increased travel demands during placements	None identified
Cheung et al. (2016) **Country:** Hong Kong, China **Quality Score:** 100%	**Aim:** To examine the prevalence of depression, anxiety and stress in nursing students **Study design:** Quantitative cross‐sectional **Sample characteristics:** 661 students enrolled in a general nursing (*n* = 451, 68.2%) or mental health nursing (*n* = 210, 31.8%) Baccalaureate degree	**Sources of financial stress:** 429 (64.9%) students reported financial difficulty, although this was not defined **Alterations to mental health and wellbeing:** Moderate to extreme levels of depression (24.3%) and anxiety (39.9%) were reported Students experiencing financial stress were significantly more likely to report depressive symptoms (aOR 2.646, B = 0.973, *p* < 0.001, 95% CI: 1.78–3.93) and anxiety symptoms (aOR 2.252, B = 0.812, *p* < 0.001, 95% CI: 1.60–3.18)	Bivariate analysis between financial stress and personal support variables was not reported. Multiple linear regression analysis demonstrated that statistically significant predictors of depression symptoms also included: Poor sleep (aOR 2.035, B = 0.711, *p* = 0.001, 95% CI: 1.36–3.05) and Physical inactivity (aOR 1.626, B = 0.486, *p* = 0.017, 95% CI: 1.09–2.43) Statistically significant predictors of anxiety symptoms also included: Poor sleep (aOR 1.487, B = 0.397, *p* = 0.44, 95% CI: 1.01–2.19) and Poor diet (aOR 1.782, B = 0.578, *p* = 0.026, 95% CI: 1.07–2.96)	Bivariate analysis between financial stress and social support variables was not reported. Multiple linear regression analysis demonstrated that along with financial stress, statistically significant predictors of depression symptoms included relationship crisis with family (aOR 3.051, B = 1.116, *p* = 0.007, 95% CI: 1.35–6.88)	None identified
Kevern and Webb (2004) **Country:** England **Quality score:** 90%	**Aim:** To explore the experiences of mature women studying nursing and consider ways to address their needs **Study design:** Qualitative **Sample characteristics:** 32 mature women aged 27 to 50 years enrolled in pre‐registration program at a single institution	**Sources of financial stress:** Travel costs (petrol) Accommodation costs (rent/mortgage on primary residence) Household and personal expenses including paying for childcare Limited income (“trying to survive on a bursary”) **Alterations to mental health and wellbeing:** Feelings of tension, conflict and guilt related to competing priorities	Students regarded themselves as highly motivated with their life experience and maturity contributing to time management and multi‐tasking skills that helped them balance their personal and academic obligations. Perceptions of having more to lose both heightened their fear of failure and motivated them to succeed	Students received instrumental support from their older children and friends who provided childcare, but they found it difficult to reciprocate which fuelled feelings of guilt. They also received emotional support through friendships with other mature women in the course which were precipitated by an awareness of each other's needs	Students received a bursary; however, this was insufficient to meet financial obligations and often needed to be supplemented with paid employment. Students felt that institutions were not doing enough to support mature students
Ogunsiji and Wilkes (2004) **Country:** Australia **Quality score:** 80%	**Aim:** To explore the lived experiences of single mothers studying nursing **Study design:** Qualitative hermeneutic phenomenology **Sample characteristics:** Five single mothers enrolled in an undergraduate nursing program at a single institution	**Sources of financial stress:** Limited income (part‐time work, occasional or absent child support payments and social welfare payments); and Household and personal expenses **Alterations to mental health and wellbeing:** Students reported feeling frustrated, worn out, tired, exhausted, incompetent, incapable, inadequate and depressed	Despite the struggle and challenges associated with being the sole provider, the students' discipline, time management and prioritisation skills, and positive outlook were considered valuable. Feelings of achievement and joy were validated by their own and their children's successes	Students received instrumental support from other people who facilitated pick up and drop off of children. They also received emotional support from their peers by forming new friendships with people with children or other single mothers with mutual understanding of circumstances. This helped reduce feelings of isolation and loneliness	Students received financial support through government‐funded social welfare payments. These payments were inadequate to meet the daily needs of themselves or their children and any paid employment reduced them, further contributing to financial stress
Santos et al. (2020) **Country:** Brazil **Quality score:** 100%	**Aim:** To explore the association between sleep quality and smoking, stress, sociodemographic and academic variables in nursing students **Study design:** Quantitative cross‐sectional **Sample characteristics:** 286 undergraduate students enrolled in a public higher education institution	**Sources of financial stress:** 186 (65%) respondents were not satisfied with their monthly family income 135 students reported their monthly family income was less than four times the minimum wage, 101 students reported it was between four and seven times the minimum wage, and 50 students reported in was more than seven times the minimum wage **Alterations to mental health and wellbeing:** 165 respondents who were not satisfied with their income also reported poor sleep quality although it was not statistically significant (*p* = 0.115) In the multivariate analysis, students whose monthly family income was below four minimum wages had a 20% increase in poor sleep quality compared to those with income greater than seven minimum wages (PR = 1.20; 95% CI 1.01; 1.43)	No personal supports identified	Having a monthly family income greater than seven times the minimum wage was associated with better sleep quality, implying greater instrumental support	No systemic supports identified
Silva et al. (2021) **Country:** Portugal **Quality score:** 87.5%	**Aim:** To analyse the relationship between nursing students' psychological wellbeing and coping strategies during the COVID‐19 quarantine **Study design:** Quantitative cross‐sectional **Sample characteristics:** 136 students enrolled in an undergraduate nursing program	**Sources of financial stress:** 64 (46.4%) students reported a reduction in household income due to the pandemic, which was not defined **Alterations to mental health and wellbeing:** Overall, students who experienced a decrease in household income reported significantly lower psychological wellbeing scores compared to those who did not (t = −2.3, *p* = 0.024)	There was a statistically significant difference in self‐esteem between students who experienced a decrease in household income and those who did not (t = −2.1, *p* = 0.042) There were no statistically significant differences in the coping strategies between groups	There was a statistically significant difference in sociability between students who experienced a decrease in household income and those who did not (*t* = −2.7, *p* = 0.008) Students with reduced household income were not treated as a distinct subgroup in the bivariate analysis of psychological wellbeing subscales and coping strategies	No systemic supports identified
Tiga‐Loza et al. (2024) **Country:** Columbia **Quality score:** 100%	**Aim:** To identify the factors associated with mental health disturbances among nursing students during the COVID‐19 pandemic **Study design:** Quantitative cross‐sectional **Sample characteristics:** 302 students enrolled in nursing programs at private universities in Columbia (*n* = 227) and Spain (*n* = 75)	**Sources of financial stress:** 144 (47.68%) respondents reported low socioeconomic status; and 159 (52.65%) respondents reported financial difficulties including job loss, death of financial supporters, and paying for college, food, housing, transportation and internet **Alterations to mental health and wellbeing:** 187 (61.92%) students exhibited a mental health disturbance, and 107 (44.4%) students exhibited clinically relevant symptoms of PTSD In the bivariate analysis, prevalence of mental health disturbance was significantly greater for students who had difficulties paying for food (PR = 1.48; 95% CI 1.26, 1.75; *p* < 0.001) and housing (PR = 1.46; 95% CI 1.22, 1.75; *p* = 0.002) In the multivariate analysis, the prevalence of mental health disturbance was significantly greater for students who had difficulties paying for food (PR = 1.35; 95% CI 1.09, 1.67; *p* = 0.007)	No personal supports identified	Bivariate analysis between financial stress and social support variables was not reported. The multiple logistic regression model showed that along with difficulty paying for food, the prevalence of mental health disturbance increased significantly for students with: Moderate family dysfunction (PR = 1.77; 95% CI 1.15, 2.73; *p* = 0.01); and Relationship breakup or loss (PR = 1.27; 95% CI 1.02, 1.59; *p* = 0.035)	No systemic supports identified
Usher et al. (2005) **Country:** Australia **Quality score:** 80%	**Aim:** To explore the challenges faced by Indigenous nursing students and strategies that aided their progression **Study design:** Qualitative descriptive **Sample characteristics:** 22 Indigenous students from Australian universities	**Sources of financial stress:** Existing financial disadvantage Cultural expectation to share money with extended family; and Course‐related expenses including textbooks, uniforms and clinical placements **Alterations to mental health and wellbeing:** Students reported feelings of anguish caused by not having enough money and characterised their experience as struggling to survive	Self‐determination and fear of failure acted as motivators to taking full advantage of the available supports, resulting in persistence and success. These personal factors were seen as crucial for navigating and responding to challenges as they arose	Family and friends provided emotional and sometimes instrumental support through financial aid and childcare	Students received financial support through government‐funded social welfare payments and scholarships. They were also provided access to equipment and resources by University Indigenous support units. Institutional supports were reported to vary widely in their availability and quality, however, in any case, were perceived as helpful in alleviating financial stress and promoting retention
Usher et al. (2022) **Country:** Australia **Quality score:** 75%	**Aim:** To investigate the financial challenges related to mandatory work‐integrated learning placements in nursing **Study design:** Quantitative and qualitative cross‐sectional **Sample characteristics:** 2359 students enrolled in a pre‐registration Bachelors (*n* = 2232) or Masters (*n* = 76) program who had attended at least one work‐integrated learning placement	**Sources of financial stress:** Travel costs (private and public transport) Accommodation costs (rent/mortgage on primary residence and temporary lodgings during placement) Loss of income where placement impacted employment; and Household and personal expenses including utilities, childcare/school fees, medical fees, groceries and loan repayments **Alterations to mental health and wellbeing:** 1173 students (61%) agreed or strongly agreed that financial stress during their most recent placement affected their health and wellbeing as reduced enjoyment of the placement, exhaustion and depression	Students increased their working hours between placements to accrue savings and leave entitlements. They also adopted cost minimisation strategies by sourcing cheaper travel (locations with low or no‐cost parking and walking), accommodation (staying with family or friends), and meal alternatives (preparing own meals, eating less, or purchasing cheaper groceries). Alternatives were still costly and/or at the detriment of nutritional value	Students received instrumental support as personal loans from family, accommodation from family and friends, and by travelling with other students. These supports offset financial stress, albeit not entirely	Only 39% of students received financial support in the form of government‐funded social welfare payments, scholarships, or allowances Those in receipt of financial support experienced delays, with 70% of scholarships/allowances and 56% of university‐based funds provided 1 month or more after placement, limiting their effectiveness

### 4.3 Risk of Bias

A quality assessment score was calculated for each study and is presented in Table 3. A score greater than 80% was considered high quality, a score between 60% and 79% was considered moderate quality, and a score less than 59% was considered poor quality (Villarosa et al. 2019).

**TABLE 3:** Critical appraisal of studies included in this review.

Study	Andrew et al. (2022)	Kevern and Webb (2004)	Ogunsiji and Wilkes (2004)	Usher et al. (2005)	Cheung et al. (2016)	Santos et al. (2020)	Silva et al. (2021)	Tiga‐Loza et al. (2024)	Usher et al. (2022)
Study type	Qualitative	Qualitative	Qualitative	Qualitative	Analytical Cross‐Sectional	Analytical Cross‐Sectional	Analytical Cross‐Sectional	Analytical Cross‐Sectional	Analytical Cross‐Sectional
Is there congruity between the stated philosophical perspective and the research methodology?	Yes	Yes	Yes	Yes	—	—	—	—	—
Is there congruity between the research methodology and the research question or objectives?	Yes	Yes	Yes	Yes	—	—	—	—	—
Is there congruity between the research methodology and the methods used to collect data?	Yes	Yes	Yes	Yes	—	—	—	—	—
Is there congruity between the research methodology and the representation and analysis of data?	Yes	Yes	Yes	Yes	—	—	—	—	—
Is there congruity between the research methodology and the interpretation of results?	Yes	Yes	Yes	Yes	—	—	—	—	—
Is there a statement locating the researcher culturally or theoretically?	No	Yes	No	No	—	—	—	—	—
Is the influence of the researcher on the research, and vice‐versa, addressed?	No	Unclear	No	No	—	—	—	—	—
Are participants, and their voices, adequately represented?	Yes	Yes	Yes	Yes	—	—	—	—	—
Is the research ethical according to current criteria, or, for recent studies, is there evidence of ethical approval by an appropriate body?	Yes	Yes	Yes	Yes	—	—	—	—	—
Do the conclusions drawn in the research report flow from the analysis, or interpretation, of the data?	Yes	Yes	Yes	Yes	—	—	—	—	—
Were the criteria for inclusion in the sample clearly defined?	—	—	—	—	Yes	Yes	Yes	Yes	Yes
Were the study subjects and the setting described in detail?	—	—	—	—	Yes	Yes	Yes	Yes	Yes
Was the exposure measured in a valid and reliable way?	—	—	—	—	Yes	Yes	Yes	Yes	Yes
Were objective, standard criteria used for measurement of the condition?	—	—	—	—	Yes	Yes	Yes	Yes	No
Were confounding factors identified?	—	—	—	—	Yes	Yes	Yes	Yes	Yes
Were strategies to deal with confounding factors stated?	—	—	—	—	Yes	Yes	No	Yes	No
Were the outcomes measured in a valid and reliable way?	—	—	—	—	Yes	Yes	Yes	Yes	Yes
Was appropriate statistical analysis used?	—	—	—	—	Yes	Yes	Yes	Yes	Yes
Total (%)	80	90	80	80	100	100	87.5	100	75

All four qualitative studies (Andrew et al. 2022; Kevern and Webb 2004; Ogunsiji and Wilkes 2004; Usher et al. 2005) were assessed as high quality, demonstrating strong congruity between their philosophical perspectives, study design, data collection, and the representation of participants' voices. However, a lack of reflexivity regarding researchers' positionality and its potential influence on data analysis was a consistent limitation. Only Kevern and Webb (2004) explicitly acknowledged power imbalances between researchers and participants, while the other studies did not address researcher influence, potentially limiting the transparency and depth of findings. Of the analytical cross‐sectional studies, four (Cheung et al. 2016; Santos et al. 2020; Silva et al. 2021; Tiga‐Loza et al. 2024) were assessed as high quality due to their use of reliable measurements and appropriate statistical analysis. Usher et al. (2022) was assessed as moderate quality as it used survey questions derived from prior studies without reporting validity measures and did not control for confounding factors during data analysis. This may have introduced bias, particularly when interpreting associations between variables.

The authors also acknowledge that the interconnected nature of financial stress with demographic and contextual factors posed a challenge during data extraction and synthesis, particularly in isolating financial stress from confounding factors. To address the risk of bias, all studies included in the review were assessed independently by multiple authors using standardised appraisal tools to ensure consistent and objective evaluation. A systematic approach was applied to data extraction where only data related to experiences of financial stress were included for analysis. Regular meetings between authors facilitated critical discussion, reflexivity, and consistent interpretation of findings.

### 4.4 Impacts on Mental Health and Wellbeing

Financial stress was consistently linked to emotional distress, characterised by intense, negative feelings and psychological states. In a survey of Chinese nursing students, Cheung et al. (2016) identified moderate to severe depression and anxiety symptoms, which, following further analysis, indicated a more than twofold increase in the incidence of symptoms for those experiencing financial difficulty. Similar emotional distress was reported in an Australian study, where 61% of students surveyed agreed that financial stress during placement affected their health and wellbeing (Usher et al. 2022). A Columbian study highlighted the impact of financial stress during the COVID‐19 pandemic, with 61.92% of students identified as having a mental health disturbance, and 44.4% of students exhibiting symptoms of post‐traumatic stress (Tiga‐Loza et al. 2024).

The 46.4% of students who reported reduced household income in the study by Silva et al. (2021) demonstrated worse overall psychological wellbeing scores, specifically affecting their sociability and self‐esteem. Depressive experiences were exacerbated by pressure from family responsibilities, as described by Ogunsiji and Wilkes (2004). Single mothers reported being frustrated by their lack of financial resources, particularly when it impacted their ability to support their children's participation in extracurricular activities, which led to feelings of incompetence and inadequacy (Ogunsiji and Wilkes 2004). Kevern and Webb (2004) also noted feelings of tension, conflict and guilt for women aged over 25 years, which largely stemmed from dual pressures of managing financial responsibilities and maintaining social relationships.

The impact on mental health and wellbeing extends beyond emotional distress, profoundly affecting students' quality of life. Usher et al. (2005) described students' feelings of anguish related to their lack of financial resources and their “continual struggle to survive”, underscoring the persistent negative impact of financial stress. Usher et al. (2022) also identified that financial stress detracted from students' clinical placement experiences, with more than half the students reporting that financial stress made it difficult to enjoy their most recent placement. Tiredness and exhaustion were other common complaints which students attributed to working additional hours to generate income (Andrew et al. 2022) and juggling competing personal, academic and clinical priorities (Ogunsiji and Wilkes 2004; Usher et al. 2022). Tiredness was also attributed to poor sleep quality, which was linked with financial stress, as reported by both Santos et al. (2020) and Cheung et al. (2016). Moreover, Cheung et al. (2016) identified poor sleep quality as a factor contributing to the prevalence of depression and anxiety symptoms, illustrating a cyclical relationship between emotional distress and reduced quality of life.

### 4.5 Sources of Financial Stress

Financial stress for nursing students was multifaceted, arising simultaneously from a range of sources across their personal, academic, and clinical domains. Despite including only credit card debt as an indicator of financial stress, nearly two thirds of the respondents in the study by Cheung et al. (2016) reported experiencing financial difficulty highlighting its widespread prevalence among nursing students. Additionally, almost half of the respondents in Silva et al. (2021) reported a reduction in household income during the COVID‐19 pandemic, illustrating the circumstantial nature of financial stress. In the seven remaining studies, the sources of financial stress were well‐defined and impacted a greater proportion of participants, underscoring the complexity of nursing students' experiences.

Limited income featured as a prominent driver of financial stress, often resulting from part‐time or casual employment (Andrew et al. 2022; Ogunsiji and Wilkes 2004) or low baseline socioeconomic status (Tiga‐Loza et al. 2024). This was not assuaged in households where partners were also in low paid occupations (Andrew et al. 2022) or when family income remained near minimum wage thresholds (Santos et al. 2020). Cultural background also exacerbated the financial stress experienced by Indigenous students who encountered persistent systemic disadvantages (Usher et al. 2005). For this cohort of students, financial stress was compounded by a unique cultural practice within Indigenous communities, which involves an expectation to share financial resources with extended family members (Usher et al. 2005).

Income disruptions were another source of financial stress, often caused by reducing paid working hours during mandatory, unpaid clinical placements (Andrew et al. 2022; Usher et al. 2022). The dissolution of spousal relationships and loss of a second income stream further depleted resources (Andrew et al. 2022). Despite these disruptions, regular expenses and the general cost‐of‐living continued to contribute to financial stress. Household and personal expenses such as rent, mortgage, or other loan repayments, were notable sources of financial stress (Kevern and Webb 2004; Tiga‐Loza et al. 2024; Usher et al. 2022). Childcare fees were a significant stressor, especially for those juggling parenting responsibilities with their studies (Kevern and Webb 2004; Ogunsiji and Wilkes 2004; Usher et al. 2022, 2005). Spending on utilities and groceries further strained already limited resources (Ogunsiji and Wilkes 2004; Tiga‐Loza et al. 2024; Usher et al. 2022).

Additionally, course‐related expenses such as textbooks, equipment, and uniforms added to the burden (Usher et al. 2005). Travel expenses, whether for public transport or private vehicles, were exacerbated by the distance between homes and clinical placements (Kevern and Webb 2004; Usher et al. 2022). When considerable distances required temporary accommodation closer to the clinical placement facility, which was common in rural and remote areas, these costs added another layer of financial burden (Usher et al. 2022).

### 4.6 Sources and Effectiveness of Support

#### 4.6.1 Personal Support

Nursing students in the included studies reported personal support in two main categories. The first was tenacity, which comprised all the personal qualities required to navigate and respond to challenges. Among these qualities were discipline, persistence, determination, and a positive outlook, which were exemplified by Indigenous students (Usher et al. 2005), single mothers (Ogunsiji and Wilkes 2004), and women aged over 25 years (Kevern and Webb 2004). Interestingly, the fear of failure acted as a motivator for Indigenous students (Usher et al. 2005) and women aged over 25 years (Kevern and Webb 2004) as their perception of having more to lose fuelled their desire to succeed. Certain skills and behaviours were associated with positive experiences; for example, time management and multitasking (Kevern and Webb 2004; Ogunsiji and Wilkes 2004). Adequate sleep, regular physical activity, and a balanced diet were also predictors of improved mental health and wellbeing (Cheung et al. 2016).

The second category was resource optimisation wherein students used strategies to leverage their assets and maximise their resources to mitigate financial stress. For example, students in two studies reported increasing their paid working hours between placements to boost their income, savings, and leave entitlements, which they later used during periods of financial stress (Andrew et al. 2022; Usher et al. 2022). However, this strategy led to a loss of downtime, negatively impacting mental health and wellbeing. Moreover, to meet financial obligations, students with dependent children (Ogunsiji and Wilkes 2004), partners with low income,: and those in broken relationships (Andrew et al. 2022) often increased their paid working hours out of necessity rather than choice.

Nursing students adopted various cost‐minimisation strategies to manage their financial constraints while studying and attending placements (Usher et al. 2022). These included finding cheaper travel options like low‐cost parking or group commuting, securing affordable accommodation by staying with family or friends, and choosing cheaper food alternatives by cooking their meals or buying less expensive groceries. Despite these efforts, they continued to experience challenges. Some students moved back with their parents but experienced increased travel costs due to longer commutes (Andrew et al. 2022), while others resorted to cheaper food alternatives, which often compromised the nutritional quality: “we only buy cheap food and eat junk meals most of the time” (Ogunsiji and Wilkes 2004, 113).

#### 4.6.2 Social Support

Social support, as defined by Zhou (2014, 6161), is “the network of resources that an individual perceives… rooted in the concepts of mutual assistance, guidance, and validation about life experiences and decisions”. Two types of social support identified among the seven studies were instrumental and emotional, with instrumental support being the most common form. Instrumental support, which describes “the various forms of tangible assistance that an individual receives in their day‐to‐day life” (Zhou 2014, 6162), reduced financial stress in two ways. The first related to the availability of financial resources, such as having a greater combined family income (Santos et al. 2020), sharing income with partners (Andrew et al. 2022), or receiving personal loans from family (Usher et al. 2022, 2005). The second concerned relief from financial obligations through various means, such as free childcare from family and friends (Kevern and Webb 2004; Ogunsiji and Wilkes 2004; Usher et al. 2005), sharing accommodation costs by living with parents (Andrew et al. 2022) or with family and friends (Usher et al. 2022), and cutting travel expenses through group commuting to placements (Usher et al. 2022).

Emotional support describes “the [intangible] support that helps to make an individual feel cared for and which serves to improve their sense of self‐worth” (Zhou 2014, 6162). The studies involving Indigenous students (Usher et al. 2005), single mothers (Ogunsiji and Wilkes 2004), and women aged over 25 years (Kevern and Webb 2004) highlighted how familial bonds and friendships were strengthened by mutual understanding of shared hardship. In turn, this lessened feelings of social isolation and loneliness (Ogunsiji and Wilkes 2004), and facilitated a sense of belonging (Usher et al. 2005). In contrast, Silva et al. (2021) found that students experiencing financial stress were significantly less sociable, while Cheung et al. (2016) reported that negative family relationships contributed to depressive symptoms. Similarly, Tiga‐Loza et al. (2024) identified that family dysfunction and relationship breakdowns contributed to mental health disturbance. Together, these findings underscore the double‐edged role of relationships as both a source of support and a potential stressor.

#### 4.6.3 Systemic Support

Systemic support for nursing students primarily took the form of financial aid, including government‐funded social welfare payments (Ogunsiji and Wilkes 2004; Usher et al. 2022, 2005), scholarships (Usher et al. 2022, 2005), bursaries (Kevern and Webb 2004), and allowances (Usher et al. 2022). Despite the availability of these financial aids, they were frequently reported to be insufficient, forcing recipients to engage in supplementary paid employment to meet their financial obligations (Kevern and Webb 2004; Ogunsiji and Wilkes 2004). This was particularly challenging for single mothers as any income they earned reciprocally reduced their social welfare payments, ultimately leaving them worse off overall (Ogunsiji and Wilkes 2004).

Indigenous students also benefited from dedicated Indigenous support services that provided access to equipment and resources, which were perceived as helpful in reducing financial stress and promoting retention (Usher et al. 2005). However, limited awareness of these supports hindered their uptake, resulting in underutilisation (Usher et al. 2022, 2005). Additionally, the timing of financial aid further reduced its effectiveness, as revealed in the study by Usher et al. (2022), where most students received their payments only after their placements had ended, providing no relief from financial stress when it was most needed.

## 5 Discussion

This review explored the direct relationship between financial stress and the mental health and wellbeing of nursing students. Although stress in this population is well documented (Chernomas and Shapiro 2013; Labrague et al. 2017, 2018; Pulido‐Martos et al. 2012), and financial stress is recognised as a contributing factor (Timmins and Kaliszer 2002), few empirical studies have examined its specific impact. Findings from this review suggest that financial stress acts as both an independent and compounding stressor—one that amplifies existing vulnerabilities and contributes to emotional distress and reduced quality of life. These effects are especially pronounced among marginalised and underserved groups including Indigenous students (Usher et al. 2022, 2005), single mothers (Ogunsiji and Wilkes 2004), women with family responsibilities (Andrew et al. 2022), and women aged over 25 years (Kevern and Webb 2004). For many of these students, financial stress is a reality even before accounting for the additional costs associated with nursing education (Andrew et al. 2022; Usher et al. 2022, 2005).

Across the included studies, three sources of financial stress were identified: personal circumstances (Andrew et al. 2022; Kevern and Webb 2004; Ogunsiji and Wilkes 2004; Santos et al. 2020; Usher et al. 2022, 2005) course‐related expenses (Andrew et al. 2022; Usher et al. 2022), and the demands of clinical placements (Andrew et al. 2022; Usher et al. 2022). The intensity and timing of stress contributed by each source varied according to students' demographic profiles and fluctuated over the course of their educational journeys. These patterns align with wider research on sociodemographic disparities in student mental health, which show that marginalised and underserved groups disproportionately experience psychological distress (De Groot et al. 2024). Caponnetto et al. (2025) similarly observed that characteristics across nursing cohorts, such as age, gender, and socioeconomic status, were largely non‐modifiable. Despite their relevance, few studies included in this review controlled for these variables, making it difficult to isolate financial stress as a distinct determinant of mental health outcomes. Further research using multivariable models is needed to interrogate these interactions.

Financial stress appeared to intensify for students managing complex personal circumstances and during periods of clinical placement (Andrew et al. 2022; Kevern and Webb 2004; Usher et al. 2022), with lasting effects on mental health and wellbeing. These findings are consistent with current evidence associating financial stress with increased anxiety, depressive symptoms, and psychological distress (Frankham et al. 2020; Talamonti et al. 2023). The stress‐vulnerability model (Zubin and Spring 1977) offers a useful framework for interpreting these outcomes. As stress accumulates, it can exceed an individual's vulnerability threshold and increase their risk of mental health deterioration. Within this model, protective factors such as social support, coping skills, or financial assistance can buffer the impact of stress and reduce the likelihood of adverse outcomes. Therefore, early and targeted interventions could serve as buffers during critical points in students' academic and clinical trajectories. This point was also emphasised by Brown and Edelmann (2000), who found that timely and adequate financial assistance significantly reduced students' stress. Nevertheless, while protective factors can moderate vulnerability (Rutter 2012), this review showed that financial stress often surpasses nursing students' coping thresholds—even when internal and external supports were available.

Protective mechanisms such as financial aid (Kevern and Webb 2004; Ogunsiji and Wilkes 2004; Usher et al. 2022, 2005), resource optimisation strategies (Andrew et al. 2022; Ogunsiji and Wilkes 2004; Usher et al. 2022), and social support (Andrew et al. 2022; Kevern and Webb 2004; Ogunsiji and Wilkes 2004; Usher et al. 2022, 2005) were commonly cited. However, the support described in these studies was largely reactive and limited in scope. In some cases, it inadvertently created new sources of financial stress. For example, students in Andrew et al. (2022) who returned home to reduce living expenses faced greater travel demands during placements. In other cases, supports offered only short‐term relief and failed to address the systemic drivers of financial stress. Most notably, financial aid often fell short of covering students' actual needs, leading many to take on additional paid work, thereby exacerbating time‐related pressures (Kevern and Webb 2004). These findings illustrate the indirect effects of financial stress where nursing students who work more than 20 h per week are at greater risk of poor academic performance and subsequent attrition (Salamonson et al. 2020). Collectively, they expose a fundamental flaw in existing support mechanisms, namely how they do not recognise the pre‐existing disadvantages shaping students' financial stress profiles nor prevent that stress from shifting across domains and compounding over time. Vulnerable students become trapped in cycles of stress and distress, with cumulative consequences for their mental health and educational outcomes.

Structured employment models may offer a viable path forward by enabling students to earn income while gaining relevant clinical experience. Programs such as the Registered Undergraduate Student of Nursing (RUSON) in Australia, the Registered Nurse Degree Apprenticeship (RNDA) in the United Kingdom, and the Undergraduate Nurse Employment Demonstration Project (UNDP) in Canada exemplify this approach. Evaluations of these programs report improvements in confidence, communication, and readiness for professional practice (Kenny et al. 2019; Lindsay et al. 2023); increased empowerment and loyalty to employers (Derbyshire et al. 2024); and a reduced need for post‐graduation orientation (Gamroth et al. 2006). However, despite their promise, these programs do not yet provide empirical evidence directly linking their implementation to reduced financial stress or improved mental health outcomes, again highlighting the current knowledge gap. Caution is also warranted, as scholars have noted that models resembling traditional apprenticeships may risk compromising the academic and professional status the discipline has fought to establish (O'Connor 2007). When students are positioned as essential members of the workforce rather than supernumerary learners, the line between education and vocational training becomes blurred.

Underlying many of these challenges is a broader issue: nursing students often lack the financial literacy to effectively navigate periods of financial stress. Without these skills, they become dependent on the fleeting and ineffectual supports identified in this review. Grant‐Smith and de Zwaan (2019) advocate for the integration of financial literacy education as a way to build long‐term financial resilience. While some may argue this should occur before pursuing higher education, financial literacy should be viewed as an evolving, context‐specific capability. Foundational learning introduced in primary or secondary education could be reinforced within nursing curricula to reflect the financial realities of contemporary practice.

Finally, a truly proactive strategy would involve greater transparency about the inherent demands of nursing education. Providing this information at the point of enrolment may deter some prospective students, but it is essential for informed decision‐making. It also supports the case for more comprehensive and targeted support systems. Yet in reality, resource constraints within higher education are likely to persist, and as efforts to diversify nursing cohorts expand, so too will the number of at‐risk students. In response, nursing education must adopt an approach similar to clinical triage that prioritises limited resources based on need and targets support where it is most likely to mitigate harm. While financial stress cannot be wholly eradicated, its most damaging impacts can be mitigated through timely, tailored, and equitable action.

### 5.1 Strengths and Limitations

A key strength of this review is its focus on the direct relationship between financial stress and mental health and wellbeing. By narrowing inclusion to studies that examined this connection, the review offers a level of granularity often missing from analyses of student stress. The review also draws attention to the disproportionate burdens experienced by marginalised and underserved groups, and highlights the compounding, cyclical nature of financial stress within the context of nursing education.

Still, several limitations should be acknowledged. First, the exclusion of studies that did not report a direct relationship between financial stress and mental health may have omitted valuable insights from research exploring indirect or multifactorial associations. Limiting the search to English‐language publications also introduces potential language bias and may have excluded relevant studies from non‐English‐speaking contexts. Future reviews could capture both indirect relationships and studies published in other languages to provide further analysis of this complex phenomenon.

Methodological inconsistencies also limited the comparability of findings. Only three of the included studies used validated tools to assess mental health and wellbeing (Cheung et al. 2016; Silva et al. 2021; Tiga‐Loza et al. 2024), and only one provided an objective measurement of financial stress (Santos et al. 2020). The lack of validated instruments and standardised definitions of financial stress limited cross‐study analysis. Future research should prioritise methodological rigour by including validated instruments and clearly defining constructs.

Contextual limitations must also be considered. Indigenous students were identified as an affected group; however, this was limited to Aboriginal and Torres Strait Islander peoples in Australia. While stress among Indigenous nursing students has been reported in Canada (Van Bewer and Sawchyn 2024), the United States of America (Kahn‐John et al. 2023), and New Zealand (Foxall et al. 2017), none of these studies explored the intersection of financial stress and mental health and wellbeing. This points to a need for culturally specific research that extends beyond the Australian context.

Geographic and systemic variability across included studies also introduced interpretive challenges. Differences in nursing program structure, duration, and national funding models likely influenced the types and intensity of financial stress reported. Notably, none of the studies included in this review identified tuition fees as a source of financial stress. This may reflect the context in which the studies were conducted. Four studies took place in Australia (Ogunsiji and Wilkes 2004; Usher et al. 2022, 2005) where eligible students can access the Government‐funded Higher Education Loan Program (HELP), and one study took place in England (Kevern and Webb 2004), where students could access National Health Service‐funded bursaries at the time of publication. These contextual differences underscore the influence of policy frameworks on student financial stress and highlight the value of comparative research examining funding models across regions.

Finally, the lack of consistent reporting on participants' year of study limits insight into how financial stress evolves across the student life cycle. Since experiences are known to fluctuate depending on stage of study, this omission makes direct comparisons more difficult and may have contributed to variability in findings. Stratifying future results by program length and year level would offer more nuanced understanding of when students are most vulnerable.

## 6 Conclusion

Although financial stress is a well‐recognised challenge within nursing education, its direct impact on students' mental health and wellbeing remains underexplored. This review contributes new insights by synthesising evidence that positions financial stress as both an independent and compounding stressor—one that amplifies existing vulnerabilities and contributes to psychological distress. By isolating this relationship, the review extends the current evidence base, particularly regarding the disproportionate effects on marginalised and underserved populations. The findings emphasise the individualised and dynamic nature of financial stress, shaped by students' demographic and contextual factors. They also expose the limitations of existing support mechanisms, which are often reactive, short‐lived, and poorly aligned with the systemic drivers of financial stress. Fundamentally, the evidence reflects a growing dissonance between the ideals of nursing education and the lived experiences of those pursuing it.

Addressing this disconnect demands a shift in perspective. Rather than attributing financial vulnerability to individual shortcomings or institutional failings, there is a need to recognise the systemic conditions that perpetuate inequity. Proactive, tailored, and sustainable interventions are essential. Structured employment models, embedded financial literacy education, and transparent pre‐enrolment communication represent promising, though under‐evaluated, strategies for reform. However, without broader structural improvements, financial stress will continue to affect those least equipped to manage it, undermining both individual wellbeing and the development of a sustainable nursing workforce.

## Author Contributions

All authors have agreed on the final version and meet at least one of the following criteria (recommended by the ICMJE*): (1) Substantial contributions to conception and design, acquisition of data, or analysis and interpretation of data; (2) Drafting the article or revising it critically for important intellectual content. *http://www.icmje.org/recommendations/.

## Conflicts of Interest

The authors declare no conflicts of interest.

## Data Availability

As this is a review of existing literature, no new datasets were generated or analysed. All data sources are publicly available through the referenced publications.

## References

[jan70043-bib-0001] Andrew, L. , J. Dare , K. Robinson , and L. Costello . 2022. “Nursing Practicum Equity for a Changing Nurse Student Demographic: A Qualitative Study.” BMC Nursing 21, no. 1: Article 37. 10.1186/s12912-022-00816-2. PMC8800819 35093048

[jan70043-bib-0002] Aromataris, E. , and Z. Munn , eds. 2020. JBI Manual for Evidence Synthesis. JBI. 10.46658/JBIMES-24-01.

[jan70043-bib-0003] Boniol, M. , T. Kunjumen , T. S. Nair , A. Siyam , J. Campbell , and K. Diallo . 2022. “The Global Health Workforce Stock and Distribution in 2020 and 2030: A Threat to Equity and ‘Universal’ Health Coverage?” BMJ Global Health 7, no. 6: e009316. 10.1136/bmjgh-2022-009316. PMC9237893 35760437

[jan70043-bib-0004] Braun, V. , and V. Clarke . 2022. Thematic Analysis: A Practical Guide. SAGE Publications.

[jan70043-bib-0005] Brough, M. , I. Correa‐Velez , P. Crane , E. Johnstone , and G. Marston . 2014. Work Integrated Learning in Social Work and Human Services: An Assessment of Financial Stress Associated With Student Placements. Queensland University of Technology. http://acen.edu.au/wp‐content/uploads/2015/09/WIL‐social‐work‐hum‐services‐assessment‐financial‐stress.pdf?x83050=.

[jan70043-bib-0006] Brown, H. , and R. Edelmann . 2000. “Project 2000: A Study of Expected and Experienced Stressors and Support Reported by Students and Qualified Nurses.” Journal of Advanced Nursing 31, no. 4: 857–864. 10.1046/j.1365-2648.2000.01344.x. 10759982

[jan70043-bib-0007] Buchan, J. , and H. Catton . 2023. Recover to Rebuild: Investing in the Nursing Workforce for Health System Effectiveness. International Council of Nurses. https://www.icn.ch/resources/publications‐and‐reports/recover‐rebuild.

[jan70043-bib-0008] Canzan, F. , L. Saiani , E. Mezzalira , E. Allegrini , A. Caliaro , and E. Ambrosi . 2022. “Why Do Nursing Students Leave Bachelor Program? Findings From a Qualitative Descriptive Study.” BMC Nursing 21, no. 1: 71. 10.1186/s12912-022-00851-z. 35351118 PMC8966353

[jan70043-bib-0009] Caponnetto, V. , E. Voltarel , V. Masotta , L. Lancia , C. Petrucci , and A. Dante . 2025. “Unveiling the Keys to Success: Insights From a Phenomenological Study on Recent Nursing Graduates.” Nurse Education Today 144: 106465. 10.1016/j.nedt.2024.106465. 39461171

[jan70043-bib-0010] Chan, Z. C. Y. , W. Y. Cheng , M. K. Fong , et al. 2019. “Curriculum Design and Attrition Among Undergraduate Nursing Students: A Systematic Review.” Nurse Education Today 74: 41–53. 10.1016/j.nedt.2018.11.024. 30580180

[jan70043-bib-0011] Chernomas, W. M. , and C. Shapiro . 2013. “Stress, Depression, and Anxiety Among Undergraduate Nursing Students.” International Journal of Nursing Education Scholarship 10, no. 1: 255–266. 10.1515/ijnes-2012-0032. 24200536

[jan70043-bib-0012] Cheung, T. , S. Y. Wong , K. Y. Wong , et al. 2016. “Depression, Anxiety and Symptoms of Stress Among Baccalaureate Nursing Students in Hong Kong: A Cross‐Sectional Study.” International Journal of Environmental Research and Public Health 13, no. 8: 1. 10.3390/ijerph13080779. PMC4997465 27527192

[jan70043-bib-0013] De Groot, K. , S. M. Wieman , J. W. Van Strien , and O. Lindemann . 2024. “To Each Their Own: Sociodemographic Disparities in Student Mental Health.” Frontiers in Education 9: 1391067. 10.3389/feduc.2024.1391067.

[jan70043-bib-0014] Dean, E. 2023. “What's the Job Costing You Where You Live in the UK?: Childcare, Housing, Transport and Utility Costs Make It Hard for Many Nurses and Students to Stick With the Profession, and the Financial Burden Varies by Region.” Nursing Standard (Royal College of Nursing (Great Britain). 1987) 38, no. 6: 19–22. 10.7748/ns.38.6.19.s12.

[jan70043-bib-0015] Department of Education . 2024. “Australian Universities Accord Final Report.” https://www.education.gov.au/australian‐universities‐accord/resources/final‐report.

[jan70043-bib-0016] Derbyshire, J. , D. Porteous , K. Corder , B. Foggo , and A. Steven . 2024. “Investigating the Processes and Influences Involved in the Transformational Journeys of Registered Nurse Degree Apprentices: A Realist Informed Qualitative Study.” Nurse Education in Practice 74: 103834. 10.1016/j.nepr.2023.103834. 38039711

[jan70043-bib-0017] Evans, C. , and A. Bonner . 2023. Combatting Placement Poverty. Griffith University. https://news.griffith.edu.au/2023/10/17/combatting‐placement‐poverty/.

[jan70043-bib-0018] Fedele, R. 2024. “Placement Poverty: Why Nursing and Midwifery Students Must Be Paid for Placement.” Australian Nursing and Midwifery Journal 27, no. 4: 8–12. https://anmj.org.au/placement‐poverty‐why‐nursing‐and‐midwifery‐students‐must‐be‐paid‐for‐clinical‐placements/.

[jan70043-bib-0019] Foxall, D. , R. Forrest , and S. Meyer . 2017. “Māori Nurses' Experiences of the Nursing Entry to Practice Transition Programme.” AlterNative: An International Journal of Indigenous Peoples 13, no. 4: 246–255. 10.1177/1177180117729853.

[jan70043-bib-0066] Frankham, C. , T. Richardson , and N. Maguire . 2020. “Psychological Factors Associated With Financial Hardship and Mental Health: A Systematic Review.” Clinical Psychology Review 77: 101832. 10.1016/j.cpr.2020.101832. 32088498

[jan70043-bib-0020] Frantzen, K. K. , and M. D. Fetters . 2016. “Meta‐Integration for Synthesizing Data in a Systematic Mixed Studies Review: Insights From Research on Autism Spectrum Disorder.” Quality & Quantity 50, no. 5: 2251–2277. 10.1007/s11135-015-0261-6.

[jan70043-bib-0021] Gamroth, L. , C. Budgen , and M. Lougheed . 2006. “Feasibility and Outcomes of Paid Undergraduate Student Nurse Positions.” Nursing Leadership 19, no. 3: e1. 10.12927/cjnl.2006.19032. 19830923

[jan70043-bib-0022] Grant‐Smith, D. , and L. de Zwaan . 2019. “Don't Spend, Eat Less, Save More: Responses to the Financial Stress Experienced by Nursing Students During Unpaid Clinical Placements.” Nurse Education in Practice 35: 1–6. 10.1016/j.nepr.2018.12.005. 30616068

[jan70043-bib-0023] Health Education England . 2018. “RePAIR: Reducing Pre‐Registration Attrition and Improving Retention Report.” https://www.hee.nhs.uk/sites/default/files/documents/RePAIR%20Report%202018_FINAL_0.pdf.

[jan70043-bib-0024] International Council of Nurses . 2023. “International Nurses Day 2023 Report—Our Nurses. Our Future.” https://www.icn.ch/resources/publications‐and‐reports/international‐nurses‐day‐2023‐report‐our‐nurses‐our‐future.

[jan70043-bib-0025] Jefferys, M. R. 2012. Nursing Student Retention: Understanding the Process and Making a Difference. 2nd ed. Springer Publishing Company.

[jan70043-bib-0026] Jeffreys, M. R. 2022. “Nursing Universal Retention and Success (NURS) Model: A Holistic, Discipline‐Focused Framework.” Journal of College Student Retention: Research, Theory & Practice 24, no. 3: 650–675. 10.1177/1521025120939254.

[jan70043-bib-0027] Kahn‐John, M. , R. Eddie , and A. Slaven . 2023. “Culturally Safe Mentoring for American Indian Nursing Students.” Creative Nursing 29, no. 4: 367–373. 10.1177/10784535231216465. 38031406

[jan70043-bib-0028] Kenny, A. , V. Dickson‐Swift , C. Phillips , N. DeVecchi , Y. Masood , and B. Hodge . 2019. Final Report: Evaluation of Registered Undergraduate Student of Nursing (RUSON) Pilot Program. La Trobe University.

[jan70043-bib-0029] Kevern, J. , and C. Webb . 2004. “Mature Women's Experiences of Preregistration Nurse Education.” Journal of Advanced Nursing 45, no. 3: 297–306. 10.1046/j.1365-2648.2003.02890.x. 14720247

[jan70043-bib-0030] Labrague, L. J. , D. M. McEnroe‐Petitte , D. Gloe , L. Thomas , I. V. Papathanasiou , and K. Tsaras . 2017. “A Literature Review on Stress and Coping Strategies in Nursing Students.” Journal of Mental Health 26, no. 5: 471–480. 10.1080/09638237.2016.1244721. 27960598

[jan70043-bib-0031] Labrague, L. J. , D. M. McEnroe‐Petitte , I. V. Papathanasiou , et al. 2018. “Stress and Coping Strategies Among Nursing Students: An International Study.” Journal of Mental Health 27, no. 5: 402–408. 10.1080/09638237.2017.1417552. 29261007

[jan70043-bib-0032] Lindsay, D. J. , T. A. Ahern , M. K. Pardon , M. T. McAuliffe , and S. G. Rannard . 2023. “Student Employment Models for Undergraduate Nurses and Midwives in Australia: A Scoping Review.” SAGE Open Nursing 9: 1–9. 10.1177/23779608231186026. PMC10328162 37425286

[jan70043-bib-0033] Lloyd, J. 2024. “Undergraduates and Poverty Placement.” Australian Nursing and Midwifery Journal 28, no. 5: 12.

[jan70043-bib-0034] Lockwood, C. , Z. Munn , and K. Porritt . 2015. “Qualitative Research Synthesis: Methodological Guidance for Systematic Reviewers Utilizing Meta‐Aggregation.” International Journal of Evidence‐Based Healthcare 13, no. 3: 179–187. 10.1097/XEB.0000000000000062. 26262565

[jan70043-bib-0035] Lockwood, C. , K. Porritt , Z. Munn , et al. 2024. “Systematic Reviews of Qualitative Evidence.” In JBI Manual for Evidence Synthesis, edited by E. Aromataris , C. Lockwood , K. Porritt , B. Pilla , and Z. Jordan . JBI. 10.46658/JBIMES-24-02.

[jan70043-bib-0036] Longmore, M. 2023. “‘Do You Live or Do You Try and Finish This Degree?’—Tauira Share the Personal Toll of Trying to Become a Nurse.” Kaitiaki Nursing New Zealand (August 2023): 1–5. https://kaitiaki.org.nz/article/do‐you‐live‐or‐do‐you‐try‐and‐finish‐this‐degree‐tauira‐share‐the‐personal‐toll‐of‐trying‐to‐become‐a‐nurse/.

[jan70043-bib-0037] Longmore, M. 2024. “Financial Support for Nursing Students ‘The Way to Go,’ Says New Head of Nelson Nursing School.” Kaitiaki Nursing New Zealand (May 2024): 1–4. https://kaitiaki.org.nz/article/financial‐support‐for‐nursing‐students‐the‐way‐to‐go‐says‐new‐head‐of‐nelson‐nursing‐school/.

[jan70043-bib-0038] Mazzotta, R. , A. Durante , V. Bressan , et al. 2024. “Perceptions of Nursing Staff and Students Regarding Attrition: A Qualitative Study.” International Journal of Nursing Education Scholarship 21, no. 1: 20230081. 10.1515/ijnes-2023-0081. 38354280

[jan70043-bib-0039] McCloud, T. , and D. Bann . 2019. “Financial Stress and Mental Health Among Higher Education Students in the UK up to 2018: Rapid Review of Evidence.” Journal of Epidemiology and Community Health 73, no. 10: 977–984. 10.1136/jech-2019-212154. 31406015 PMC6817692

[jan70043-bib-0040] Moola, S. , Z. Munn , C. Tufanaru , et al. 2024. “Systematic Reviews of Etiology and Risk.” In JBI Manual for Evidence Synthesis, edited by E. Aromataris , C. Lockwood , K. Porritt , B. Pilla , and Z. Jordan . JBI. 10.46658/JBIMES-24-06.

[jan70043-bib-0041] Moore, A. , A. Nguyen , S. Rivas , A. Bany‐Mohammed , J. Majeika , and L. Martinez . 2021. “A Qualitative Examination of the Impacts of Financial Stress on College Students' Well‐Being: Insights From a Large, Private Institution.” SAGE Open Medicine 9: 1–8. 10.1177/20503121211018122. PMC8141976 34094560

[jan70043-bib-0042] Moran, L. , T. Capper , M. Gupta , S. Meedya , and S. Mendez . 2024. “Financial Hardship and Australian Midwifery Students: A Scoping Review and Thematic Analysis.” Women and Birth: Journal of the Australian College of Midwives 37, no. 5: 101640. 10.1016/j.wombi.2024.101640. 38959594

[jan70043-bib-0043] Morton, P. G. 1983. “The Financial Distress of Higher Education: Impact on Nursing.” Image—The Journal of Nursing Scholarship 15, no. 4: 102–106. 10.1111/j.1547-5069.1983.tb01367.x. 6557993

[jan70043-bib-0044] Mulvogue, J. , C. Ryan , S. Hunt , M. Cross , and M. Cleary . 2023. “Promoting Positive Outcomes in Higher Education: Supporting Undergraduate Student Mental Health and Well‐Being.” Issues in Mental Health Nursing 44, no. 7: 673–677. 10.1080/01612840.2022.2116136. 36049210

[jan70043-bib-0045] O'Connor, S. J. 2007. “Developing Professional Habitus: A Bernsteinian Analysis of the Modern Nurse Apprenticeship.” Nurse Education Today 27, no. 7: 748–754. 10.1016/j.nedt.2006.10.008. 17134794

[jan70043-bib-0046] Ogunsiji, O. , and L. Wilkes . 2004. “Managing Family Life While Studying: Single Mothers' Lived Experience of Being Students in a Nursing Program.” Contemporary Nurse 18, no. 1–2: 108–123. 10.5172/conu.18.1-2.108. 15729803

[jan70043-bib-0047] Page, M. J. , J. E. McKenzie , P. M. Bossuyt , et al. 2021. “The PRISMA 2020 Statement: An Updated Guideline for Reporting Systematic Reviews.” BMJ 372: n71. 10.1136/bmj.n71. 33782057 PMC8005924

[jan70043-bib-0048] Pulido‐Martos, M. , J. M. Augusto‐Landa , and E. Lopez‐Zafra . 2012. “Sources of Stress in Nursing Students: A Systematic Review of Quantitative Studies.” International Nursing Review 59, no. 1: 15–25. 10.1111/j.1466-7657.2011.00939.x.

[jan70043-bib-0049] Rutter, M. 2012. “Resilience as a Dynamic Concept.” Development and Psychopathology 24, no. 2: 335–344. 10.1017/S0954579412000028. 22559117

[jan70043-bib-0050] Salamonson, Y. , D. Roach , R. Crawford , et al. 2020. “The Type and Amount of Paid Work While Studying Influence Academic Performance of First Year Nursing Students: An Inception Cohort Study.” Nurse Education Today 84: 104213. 10.1016/j.nedt.2019.104213. 31698291

[jan70043-bib-0051] Santos, A. F. , F. C. Mussi , C. G. Pires , C. A. Santos , and M. A. Paim . 2020. “Sleep Quality and Associated Factors in Nursing Undergraduates.” Acta Paulista de Enfermagem 33, no. 3: 1–8. 10.37689/actaape/2020AO0144.

[jan70043-bib-0052] Silva, M. S. , G. F. Marques , A. C. Reis , et al. 2021. “Nursing Students' Psychological Well‐Being and Coping During the COVID‐19 Quarantine.” Revista de Enfermagem Referência 5, no. 8: e20211. 10.12707/rv20211.

[jan70043-bib-0065] Talamonti, D. , J. Schneider , B. Gibson , and M. Forshaw . 2023. “The impact of national and international financial crises on mental health and well‐being: a systematic review.” Journal of Mental Health 33, no. 4: 522–559. 10.1080/09638237.2023.2278104. 37934869

[jan70043-bib-0053] Tiga‐Loza, D. C. , L. B. Arboleda de Pérez , M. Á. Ramírez‐Cruz , and R. de Diego Cordero . 2024. “Factors Related to Mental Health Problems in Nursing Students: A Multicenter Study.” Revista Cuidarte 15, no. 2: 1–13. 10.15649/cuidarte.3296. PMC11807002 40114690

[jan70043-bib-0054] Timmins, F. , and M. Kaliszer . 2002. “Aspects of Nurse Education Programmes That Frequently Cause Stress to Nursing Students—Fact‐Finding Sample Survey.” Nurse Education Today 22, no. 3: 203–211. 10.1054/nedt.2001.0698. 12027601

[jan70043-bib-0055] Usher, K. , A. Fagan , J. A. Brown , et al. 2022. “The Financial Challenges for Australian Nursing Students Attending Placement‐Based Work‐Integrated Learning.” Collegian 29, no. 2: 154–160. 10.1016/j.colegn.2021.07.005.

[jan70043-bib-0056] Usher, K. , D. Lindsay , M. Miller , and A. Miller . 2005. “Challenges Faced by Indigenous Nursing Students and Strategies That Aided Their Progress in the Course: A Descriptive Study.” Contemporary Nurse 19, no. 1–2: 17–31. 10.5172/conu.19.1-2.17. 16167431

[jan70043-bib-0057] Van Bewer, V. , and M. Sawchyn . 2024. “Enhancing Nursing Education for Indigenous Students: Indigenous Nursing Students' Insights and Strategies.” Nurse Education Today 137: 106157. 10.1016/j.nedt.2024.106157. 38503250

[jan70043-bib-0058] Villarosa, A. R. , D. Maneze , L. M. Ramjan , R. Srinivas , M. Camilleri , and A. George . 2019. “The Effectiveness of Guideline Implementation Strategies in the Dental Setting: A Systematic Review.” Implementation Science 14, no. 1: 106. 10.1186/s13012-019-0954-7. 31847876 PMC6918615

[jan70043-bib-0059] Whittemore, R. , and K. Knafl . 2005. “The Integrative Review: Updated Methodology.” Journal of Advanced Nursing 52, no. 5: 546–553. 10.1111/j.1365-2648.2005.03621.x. 16268861

[jan70043-bib-0060] World Health Organization . 2020. “State of the World's Nursing 2020: Investing in Education, Jobs and Leadership.” https://fctc.who.int/publications/i/item/9789240003279.

[jan70043-bib-0061] World Health Organization . 2021. “Comprehensive Mental Health Action Plan 2013–2030.” https://www.who.int/publications/i/item/9789240031029.

[jan70043-bib-0062] Wuth, A. , and M. Cismaru . 2021. “A Conceptual and Operational Review of the Negative Financial Health Terminology and Constructs.” International Business Research 14, no. 4: 1–16. 10.5539/ibr.v14n4p1.

[jan70043-bib-0063] Zhou, E. S. 2014. “Social Support.” In Encyclopedia of Quality of Life and Well‐Being Research, edited by A. C. Michalos , 6161–6164. Springer. 10.1007/978-94-007-0753-5_2789.

[jan70043-bib-0064] Zubin, J. , and B. Spring . 1977. “Vulnerability: A New View of Schizophrenia.” Journal of Abnormal Psychology 86, no. 2: 103–126. 10.1037/0021-843X.86.2.103. 858828

